# 308. The Agency for Healthcare Research and Quality (AHRQ) Safety Program for Improving Skin Care and MDRO Prevention: The Impact of an Educational and Implementation Project in United States (US) Long-Term Care Facilities

**DOI:** 10.1093/ofid/ofaf695.104

**Published:** 2026-01-11

**Authors:** Morgan Katz, Heather Stoltzfus, Lisa L Maragakis, Kathleen Speck, Melissa A Miller, Roy Ahn, Jennifer Titus, Yue Gao, Prashila Dullabh, Lauren Squires, Leyi Lin, Caylin Andrews, Robin Jump

**Affiliations:** Johns Hopkins, Stevenson, MD; Johns Hopkins University, Baltimore, Maryland; Johns Hopkins Medicine, Baltimore, MD; Johns Hopkins, Stevenson, MD; Agency for Healthcare Research and Quality, Rockville, Maryland; NORC, Chicago, Illinois; NORC at the University of Chicago, West Kill, New York; NORC, Chicago, Illinois; NORC at the University of Chicago, West Kill, New York; NORC at the University of Chicago, West Kill, New York; Agency for Healthcare Research and Quality, Rockville, Maryland; Johns Hopkins University, Baltimore, Maryland; Division of Geriatrics, Department of Medicine, University of Pittsburgh School of Medicine, Pittsburgh, Pennsylvania

## Abstract

**Background:**

The AHRQ Safety Program for Improving Skin Care and MDRO Prevention in Long-Term Care (LTC) implemented evidence-based skin care and infection prevention (IP) strategies combined with team and safety-culture building initiatives to reduce the development of pressure injuries and prevent infection in LTC facilities in the U.S.

**Table 1:**
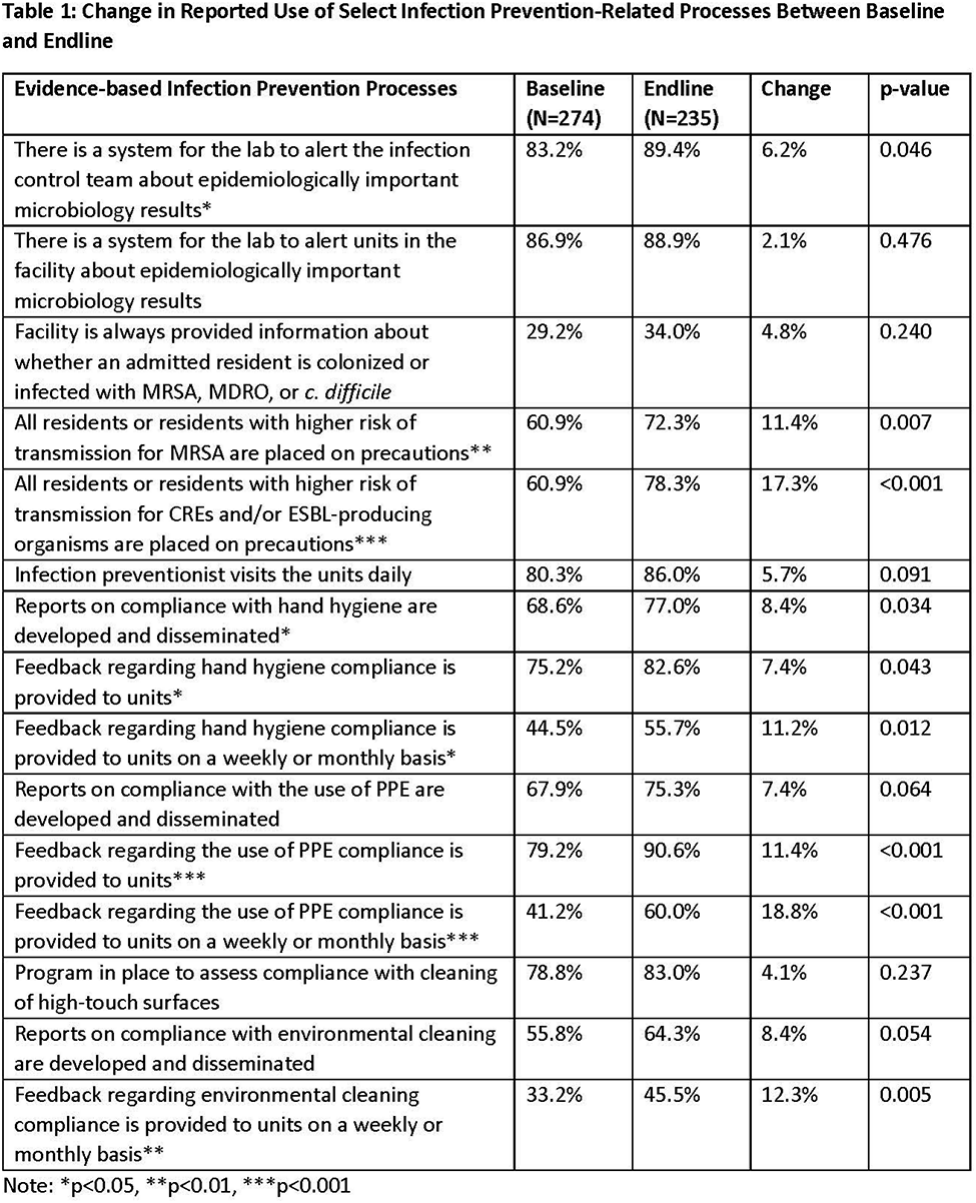
Change in Reported Use of Select Infection Prevention-Related Processes Between Baseline and Endline

**Table 2:**
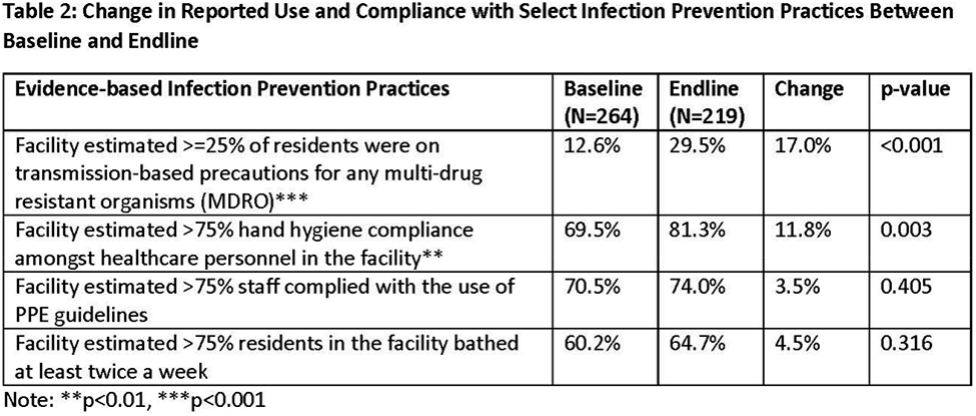
Change in Reported Use and Compliance with Select Infection Prevention Practices Between Baseline and Endline

**Methods:**

The program ran from June 2023 to November 2024. Participating facilities had access to 19 webinars paired with “teachable moments”-brief learning tools for dissemination to direct-care staff. Education focused on proper bathing and skin assessment and practices to prevent pathogen transmission (e.g., basic IP practices, using antibiotics wisely, disinfection of high-touch surfaces). Facilities were assigned an implementation adviser who provided support through monthly coaching calls.

Minimum Data Set data from June 2022 to November 2024 were obtained from the Centers for Medicare and Medicaid Services and summarized quarterly to assess pressure injuries at baseline and during the program. A linear mixed model was used to assess the change between periods. The program also collected data on the use of recommended IP-related strategies and practices.

**Table 3:**
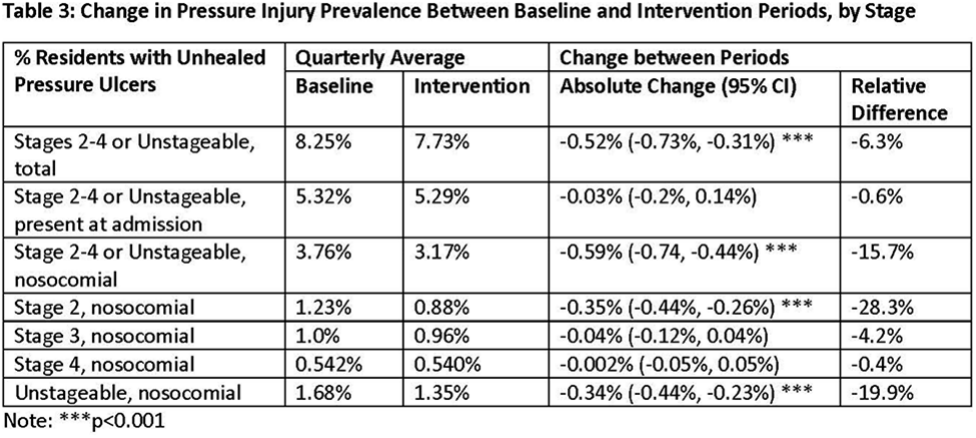
Change in Pressure Injury Prevalence Between Baseline and Intervention Periods, by Stage

**Figure 1.**
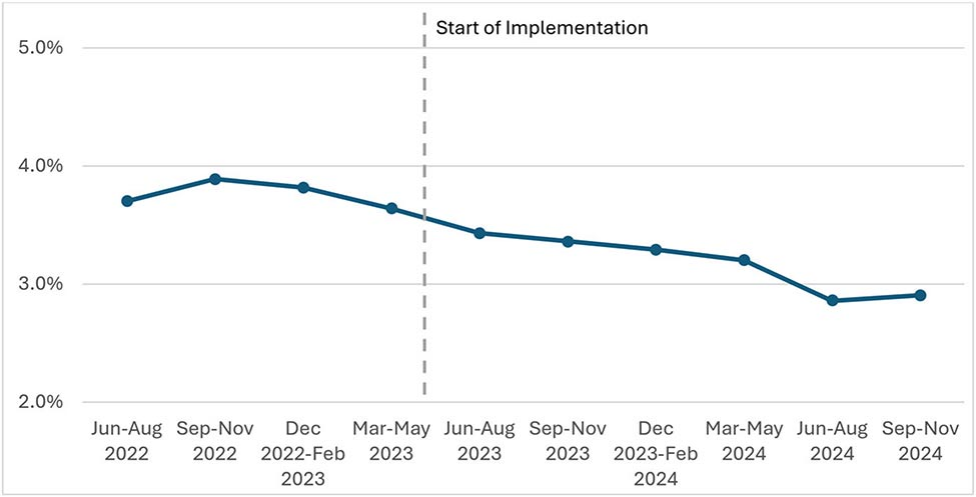
Percentage of residents with nosocomial unhealed pressure injuries at stages 2-4 or unstageable by quarters, from June-August 2022 to September-November 2024

**Results:**

309 facilities completed the Safety Program. Use of several IP strategies, such as feedback systems for hand hygiene and PPE compliance, increased during the program (Table 2). Further, use and compliance with recommended IP practices also increased after the program (Table 3). Between the baseline and intervention periods, total nosocomial stage 2-4 or unstageable ulcers decreased by 15.7% (p< 0.001, Table 3, Figure 1). Nosocomial Stage 2 and unstageable pressure injuries declined 28.3% and 19.9%, respectively (both p< 0.001). No significant changes were noted in nosocomial stage 3 (-4.2%) or stage 4 pressure injuries (-0.4%). The rate of pressure injuries present on admission was unchanged between periods.

**Conclusion:**

The AHRQ Safety Program was associated with increased use of IP strategies and practices and a significant reduction in the development of nosocomial Stage 2 and unstageable pressure ulcers among residents admitted to participating nursing homes, suggesting improvements in early detection and prevention.

**Disclosures:**

Morgan Katz, MD, MHS, Skinclique: Advisor/Consultant

